# Interferon-Induced Transmembrane Protein 3 (IFITM3) Restricts PRRSV Replication via Post-Entry Mechanisms

**DOI:** 10.3390/microorganisms13081737

**Published:** 2025-07-25

**Authors:** Pratik Katwal, Shamiq Aftab, Eric Nelson, Michael Hildreth, Shitao Li, Xiuqing Wang

**Affiliations:** 1 Department of Biology and Microbiology, South Dakota State University, Brookings, SD 57007, USA; 2 Department of Veterinary and Biomedical Sciences, South Dakota State University, Brookings, SD 57007, USA; 3 Department of Microbiology and Immunology, Tulane University, New Orleans, LA 70112, USA

**Keywords:** virus restriction factors, interferon-stimulated genes (ISGs), interferon-induced transmembrane protein 3 (IFITM3), porcine reproductive and respiratory syndrome Virus (PRRSV)

## Abstract

Interferon-induced transmembrane protein 3 (IFITM3) is a member of the family of interferon-stimulated genes (ISGs) that inhibits a diverse array of enveloped viruses which enter host cells by endocytosis. Porcine reproductive and respiratory syndrome virus (PRRSV) is an enveloped RNA virus causing significant economic losses to the swine industry. Very little is known regarding how IFITM3 restricts PRRSV. In this study, the role of IFITM3 in PRRSV infection was studied in vitro using MARC-145 cells. IFITM3 over-expression reduced PRRSV replication, while the siRNA-induced knockdown of endogenous IFITM3 increased PRRSV RNA copies and virus titers. The colocalization of the virus with IFITM3 was observed at both 3 and 24 h post infection (hpi). Quantitative analysis of confocal microscopic images showed that an average of 73% of IFITM3-expressing cells were stained positive for PRRSV at 3 hpi, while only an average of 27% of IFITM3-expressing cells were stained positive for PRRSV at 24 hpi. These findings suggest that IFITM3 may restrict PRRSV at the post-entry steps. Future studies are needed to better understand the mechanisms by which this restriction factor inhibits PRRSV.

## 1. Introduction

Restriction factors function to antagonize virus infection and can target viruses at various stages of their life cycle. Some block the cytosolic entry of enveloped viruses by either directly inhibiting virus fusion at the cell surface or by preventing fusion within the endosomes [1]. Interferon-induced transmembrane proteins (IFITMs) belong to a class of interferon-stimulated genes (ISGs) and are constitutively expressed in various cell types and potently induced upon interferon treatment [2,3]. Of the five known members of the IFITM family, IFITM1, 2, and 3 are antiviral restriction factors and inhibit viruses that either enter by direct fusion with the cell membrane (IFITM1), or by endosomal trafficking (IFITM2 and IFITM3) [4,5]. IFITM3 also inhibits virus–host membrane fusion within the endosomes [6]. Other well-studied classical ISGs such as Mx1, OAS, and PKR also play important roles in restricting virus replication [7,8,9].

The role of IFITM3 in Influenza A virus (IAV) infection has been well studied [10]. IFITM3 knockout mouse embryonic fibroblasts (MEFs) are highly susceptible to IAV infection [5]. The cytosolic release of the IAV viral ribonucleoproteins (vRNPs) is restricted by IFITM3, thereby blocking their subsequent nuclear entry [2,6]. Enveloped viruses such as IAV that are pH sensitive and require a low pH for fusion are highly restricted by IFITM3 [11]. The IFITM3 protein is expressed in the early or late endosomes and strongly inhibits viruses that enter through late endosomes. Overall, the block to virus uncoating results in the trapping of viruses in the acidic endolysosomes, within which it undergoes degradation [2]. IFITM3 expression has been shown to expand acidified endosomes as evident from colocalization with endosomal markers, chiefly Rab7 and LAMP-1 [2]. Other viruses such as West Nile virus, Vesicular stomatitis virus, etc., that fuse at pH greater than 6 are also inhibited by IFITM3 [12,13]. IFITM3 proteins inhibit a broad spectrum of enveloped RNA viruses [5,12]. Recently, IFITM3 has also been shown to restrict Zika virus, an important emerging pathogen [14].

Porcine reproductive and respiratory syndrome virus (PRRSV) is an enveloped, positive-sense, and single-stranded RNA virus. It is a highly pathogenic virus that mainly causes respiratory disease in newborn pigs and reproductive disease in sows [15]. PRRSV belongs to the order *Nidovirales* within the family *Arteriviridae* [15]. Upon initial attachment and internalization via binding to the sialoadhesin molecules, PRRSV enters into the early endosome through a clathrin-dependent pathway in clathrin-coated vesicles and is dependent on its binding to the CD163 receptor [16,17]. CD163 functions as the principal PRRSV receptor that recycles between the plasma membrane and endosomes [17,18,19]. PRRSV is known to enter cells through the endocytic pathway [16,20]. However, one study showed that PRRSV does not appear to be associated with later endosomes/lysosomes [21].

The impact of many ISGs, including the impact of IFITM3 on PRRSV replication, has not been extensively studied. One recent study has reported the antiviral role of IFITM3 in PRRSV infection [22]. Zhang et al. showed that IFITM3 did not block PRRSV entry into the endosome or lysosome, but restricted PRRSV fusion through cholesterol accumulation within the endosomal membrane [22]. These authors have further suggested that IFITM3 is incorporated into PRRSV virions, which results in reduced infectivity and cell–cell spread of PRRSV [23]. Our previous study suggested that ZMPSTE24, the downstream effector of IFITM3, restricts PRRSV replication at post-entry steps [24]. Here in this study, we have examined the role of IFITM3 in PRRSV replication in MARC-145 cells. Our data suggested that IFITM3 did not impact virus entry, but restricted PRRSV replication at post-entry steps.

## 2. Materials and Methods

### 2.1. IFITM3 Transfection in MARC-145 Cells

MARC-145 cells were cultured in a 12-well plate in DMEM medium supplemented with 10% FBS and 1% Penicillin–Streptomycin (complete DMEM medium). The 12-well plate was incubated in a humidified chamber at 37 °C with 5% CO_2_. After 24 h, cells were washed in DMEM medium and transfected with 1.25 µg of either the pQCXIP vector control (Q-series retroviral vector; Retro-X Q Vector Set) (Clontech, Cat. No. 631516; Takara Bio Inc., San Jose, CA, USA) or pQCXIP expressing the IFITM3-HA [5,25] in triplicate wells using lipofectamine 3000 Transfection kit (Invitrogen, Carlsbad, CA, USA), following the manufacturer’s protocol. The retroviral vector pQCXIP carries the puromycin-resistant gene and expresses IFITM3-HA, transfected in HEK293 packaging cells for stable expression [25]. Briefly, each plasmid DNA was diluted in DMEM medium with P3000 and mixed with Lipofectamine 3000 reagent. The transfection mix was then incubated at room temperature (RT) for 20 min, after which 200 µL of the DNA-lipid complex was added dropwise to the respective wells. After incubation for 6 h at 37 °C, the medium was replaced with complete DMEM medium. After 72 h, each of the six wells was infected with PRRSV 23,983 at an MOI of 1 for 24 h. In some experiments, MARC-145 cells were treated with 1 µM of torin1 (Cell Signaling Technology, catalog# 14379, Danvers, MA, USA) for 3 h. Then, cells together with supernatant were harvested. The supernatant and the pellets were stored separately at −80 °C until used for further analysis.

Further, MARC-145 cells cultured in two 8-chamber slides (colocalization study) or a 96-well plate (cytotoxicity study) were transfected with 0.3 µg of either pQCXIP vector control or IFITM3-HA using lipofectamine 3000 reagent.

Transfection in the 48-well plate was performed in two groups. In the first group, MARC-145 cells were transfected with 0.3 µg of either pQCXIP plasmid vector or pQCXIP IFITM3-HA in triplicate using lipofectamine 3000 reagent. The second group was transfected following the same transfection scheme (Amphotericin B treatment group).

### 2.2. siRNA-Induced Knockdown of IFITM3 in MARC-145 Cells

MARC-145 cells were cultured in a 12-well plate. Following 24 h incubation at 37 °C, cells were transfected with either negative control siRNA (Ambion, cat. no. AM4642) or IFITM3 siRNA (Life technologies, siRNA ID#s195035) at a final concentration of 40 nM per well using Lipofectamine RNAi max reagent (Invitrogen) according to the manufacturer’s protocol. Transfection was performed in triplicate wells. The siRNA was mixed with RNAi max reagent diluted in DMEM medium in a 1:1 ratio. The mixture was incubated at RT for 5 min, after which 125 µL of the siRNA complex was added dropwise to the respective wells of MARC-145 cells in DMEM medium. The 12-well plate was then incubated at 37 °C for 6 h. Then the DMEM medium was replaced with complete DMEM growth medium and incubated for 72 h. Next, the cells in each well of the 12-well plate were infected with PRRSV 23,983 at an MOI of 1 for 24 h, after which the cells were scraped and harvested with supernatant. Following centrifugation, supernatant and cell pellets were separated and stored at −80 °C.

For the cytotoxicity experiment, 1.2 µL of either negative control siRNA or IFITM3 siRNA at a final concentration of 40 nM was used for transfection in triplicate wells of a 96-well plate. Thus 25 µL of transfection mix was added dropwise to the respective wells, then incubated at 37 °C for 6 h and replaced with complete DMEM medium.

### 2.3. Western Blot Analysis

Cell pellets were resuspended with 60 µL cell lysis buffer (0.01 M Tris-HCl pH 8, 0.14 M NaCl, 0.025% NaN3, 1% Triton × −100) treated with Halt^TM^ protease and phosphatase inhibitor cocktail (Thermo Scientific, catalog# 78441, Waltham, MA, USA) at a 1:100 dilution. Each lysate sample thus obtained from the over-expression of either the pQCXIP vector or the IFITM3-HA was vortexed for 15 s. The tubes were then incubated for 1 h at 4 °C, with vortexing every 15 min. Each tube was then spun at 13,000 rpm for 1 min, after which the lysate was transferred to a new tube. Next, 20 µL of each sample was mixed with equal volumes of 2× Western blot loading buffer (treated with beta-mercaptoethanol at 1:50 dilution). Each sample was incubated at 95 °C for 5 min and then loaded into each well. Gels were run at 150 V for 1.5 h and then transferred to nitrocellulose membrane. Each membrane was incubated in blocking buffer (5% milk powder in 1× PBST or 5% BSA in 1× PBST) at room temperature (RT) for 1 h. Then the membranes were washed with 1× PBST and incubated by shaking overnight at 4 °C with respective primary antibodies. The following primary antibodies were used: anti-HA mouse monoclonal 1o Ab (6E2) (Cell Signaling Technology, catalog# 2367) at 1:1000 dilution, anti-HA mouse monoclonal antibody (HA7) (Sigma Aldrich, catalog# H3663, Saint Louis, MO, USA) at 1:5000 dilution, anti-PRRSV SR-30 1o monoclonal Ab (provided by Dr. Eric Nelson) at 1:300 dilution, anti-IFITM3 rabbit polyclonal antibody (Proteintech, catalog# 11714-1-AP, Rosemont, IL, USA) at 1:1000 dilution, anti-p-Akt (S743, Cell Signaling Technology, catalog# 9271S) at 1:1000 dilution, anti-LC3B antibody (Sigma Aldrich, catalog# L7542) at 1:2000 dilution, and mouse monoclonal anti-beta actin Ab (Sigma Aldrich, catalog# A2228) at 1:5000 dilution. On the next day, the membrane was washed with 1× PBST three times and then incubated in the dark at RT with Goat anti-Mouse IRDye^®^-conjugated secondary Ab (LI-COR, catalog# 926-32210, Lincoln, NE, USA) diluted to 1:15,000. The membrane was washed and observed using the LI-COR Odyssey Infrared Imaging System.

### 2.4. Interferon Stimulation of IFITM3 Gene in MARC-145 Cells

To study the dose-dependent response of IFN-alpha treatment on IFITM3 gene expression, a 12-well plate was seeded with MARC-145 cells and incubated for 24 h at 37 °C. Next, MARC-145 cells were treated with IFN-alpha at a concentration of either 50 U/mL, or 100 U/mL, or 500 U/mL in duplicate wells. Two wells were incubated with 1 mL of DMEM complete medium only (IFN-alpha-untreated control wells). After 12 h incubation, the medium was removed, and each well was infected with 1 MOI of PRRSV 23,983 for 24 h at 37 °C. Cells were then harvested, and the cell pellets were stored at −80 °C for RT-PCR analysis of IFITM3 gene expression and PRRSV nucleocapsid (N) gene RNA copies.

### 2.5. Immunofluorescence Assay and Flow Cytometry

Amphotericin B at a concentration of 2.5 µg/mL was added to each of the six wells of a 48-well plate transfected with either vector control plasmid pQCXIP or IFITM3-HA in triplicate. Another set of six wells transfected similarly was not treated with Amphotericin B. After incubating the plate at 37 °C for 1 h, Amphotericin B was removed. PRRSV 23,983 was added at an MOI of 1 to all wells and the plate was incubated at 37 °C for 24 h. Cells were then fixed with 80% acetone for 20 min at RT and air dried. Next, FITC-conjugated anti-PRRSV Ab specific to N protein (SDOW-17) [26] at a 1:80 dilution was added to each well and incubated at 37 °C for 1 h. The cells were washed and observed using an Olympus IX70 Inverted-Microscope equipped with epi-fluorescence and phase contrast. The cells in each well were scraped, resuspended in PBS, and analyzed by flow cytometry for calculating the mean fluorescence intensity of the FITC staining.

### 2.6. Colocalization Study Using Confocal Microscopy

For the study of the colocalization of PRRSV with endosomal markers, MARC-145 cells were cultured in three 8-chamber slides for each time point study. After 24 h, the cells were infected with PRRSV at an MOI of 10. Then at three time points—1 h, 3 h, or 6 h after infection, the cells were fixed with 3.8% formaldehyde for 10 min at RT, then washed in PBS for 5 min and treated with 0.2% Triton X-100 for 10 min, after which cells were again washed once and incubated with 5% goat serum in PBS (blocking buffer) for 1 h. Then cells were incubated with SDOW17 (FITC conjugated anti-PRRSV N) monoclonal Ab at 1:80 dilution, together with either anti-EEA1 Ab (Santa Cruz Biotechnology, sc-365652, Dallas, TX, USA) or anti-Lamp-1 Ab (Santa Cruz Biotechnology, sc-19992) conjugated to Alexa Fluor 647. The cells were washed, then mounted using ProLong Gold antifade mounting reagent (Invitrogen) and observed with an Olympus Fluoview FV1200 Laser Scanning Confocal Microscope. For each time point, five random cells were selected and then with manual thresholding, the threshold range was selected to define the area of interest distinct from background [27]. The color (yellow) threshold was adjusted to measure the area of the colocalized region (A1). Next, the threshold color was adjusted to select all five cells to obtain the area of the total five cells (A2). The percentage colocalization was calculated by multiplying the fraction (A1/A2) by 100. Mean percent colocalization was calculated for each time point from three selected areas (each with five cells).

MARC-145 cells cultured in two 8-chamber slides were transfected with either vector control pQCXIP or IFITM3-HA in duplicate wells of each slide as described earlier. For the 3 h post-infection (3 hpi) study, PRRSV 23,983 at an MOI of 4 was added to all wells except one vector control-transfected well. For 24 hpi study, the cells were infected with PRRSV at an MOI of 1. After infecting the cells with PRRSV for the respective times, the two slides were fixed using the protocol as described previously. Then the cells in each well were incubated overnight at RT with primary antibody diluted in 5% goat serum. Mouse monoclonal anti-HA primary antibody against HA-tagged IFITM3 (6E2) (Cell Signaling Technology, cat. no. 2367) was added at a dilution of 1:1600 to all wells except the uninfected vector control-transfected well to which 5% goat serum was added. On the next day, the slides were washed three times with 1× PBS and then incubated for 1 h at 37 °C with secondary antibody. To the three wells incubated with anti-Flag primary antibody, Alexa fluor 647 conjugated goat anti-mouse IgG secondary Ab (Abcam) at a 1:200 dilution was added. After 1 h of incubation, the cells were washed and incubated with 1:80 diluted anti-PRRSV FITC-conjugated Ab (SDOW17) at 37 °C for 1 h. The uninfected vector control-transfected well was incubated with Alexa fluor 647 secondary Ab only. The two slides were washed, counterstained with DAPI, and then mounted as described previously. The slides were then stored at 4 °C overnight and observed with confocal microscopy to identify any colocalization between PRRSV and IFITM3 at 3 or 24 hpi. Five different microscopic fields at 40× magnification were analyzed for the pQCXIP vector control-transfected group and the IFITM3-HA-transfected group. In each field, cells were counted by DAPI staining and then the percentage of cells staining for PRRSV (green) or IFITM3 (red) was calculated. The colocalization study was performed by counting the number of DAPI-stained cells with yellow fluorescence which represents the overlap of the emission spectra for the green FITC and red Alexa fluor 647 signals. Uninfected control cells stained with FITC-conjugated anti-PRRSV Ab were used as a control for comparing the background (green) staining with the PRRSV-infected group.

### 2.7. Cytotoxicity Assay

MARC-145 cells cultured in a 96-well plate were transfected with either the pQCXIP vector control or pQCXIP encoding IFITM3-HA or negative control siRNA (Ambion, cat. no. AM4642, Austin, TX, USA) or IFITM3 siRNA (Life technologies, siRNA ID# s195035, Carlsbad, CA, USA) in triplicate wells, as described earlier. After 72 h, each well incubated with 200 µL of DMEM growth medium was treated with 10 µL of Cell Counting Kit-8 (CCK-80 solution, Sigma, cat. no. 96992) and incubated at 37 °C for 3 h, after which absorbance was measured at 450 nm using a Synergy 2 plate reader (BioTek, Shoreline, WA, USA).

### 2.8. Real-Time Reverse Transcription PCR (RT-PCR)

Total RNA was extracted from cells using RNeasy Mini Kits (Qiagen cat. no. 74104, Germantown, MD, USA) following the manufacturer’s protocol. One µg of total RNA was reverse-transcribed using a High-Capacity cDNA Reverse Transcription Kit (Applied Biosystems, Foster City, CA, USA) under the following reaction conditions: 25 °C for 10 min, 37 °C for 120 min, and 85 °C for 5 min (Applied Biosystems GeneAmp PCR System 2400). Real-time PCR was performed by using 3 µL of the cDNA for each reaction using Brilliant II SYBR Green QPCR master mix (Agilent Technologies). Forty cycles of the following conditions were used: 95 °C for 10 min, 95 °C for 30 s, 55 °C for 30 s, and 72 °C for 30 s. Primers specific to beta-actin housekeeping gene, PRRSV N, IFITM3, and Mx1 are shown in Table 1. The reactions were run in a QuantStudio 6 Flex Real-Time PCR System (Applied Biosystems). Data analysis was performed by calculating the mean fold change in gene expression using the _ΔΔ_CT method. Housekeeping gene beta-actin was used as a reference gene for comparing the fold change between the two treatment groups [28].

### 2.9. TCID_50_ Titer

A fifty percent tissue culture infectious dose (TCID_50_) assay was performed to determine the infectious PRRSV 23,983 titer in the supernatant at 24 hpi obtained from the previously described IFITM3 over-expression or siRNA-induced knockdown experiments. TCID50 titer was determined using the Spearman–Karber method.

### 2.10. Statistical Analysis

Two-tailed Student’s *t*-tests were used for all comparisons, and *p*-values less than 0.05 (*p* < 0.05) were considered significant.

## 3. Results

### 3.1. Over-Expression of Exogenous IFITM3 Reduces PRRSV Replication

**Figure 1:**
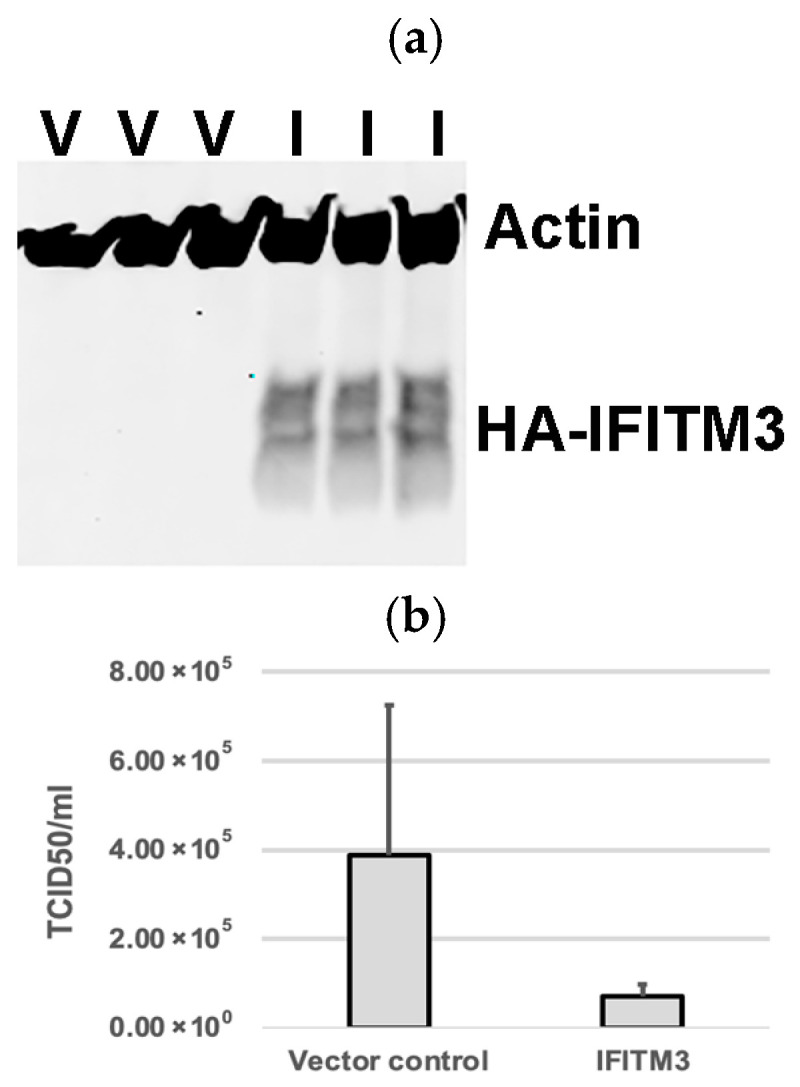

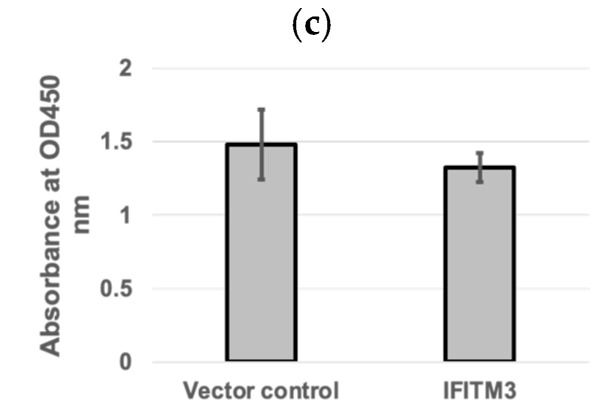
The over-expression of IFITM3 reduces PRRSV replication. (**a**) Western blotting analysis showing the expression of exogenous IFITM3 in cells transfected with a plasmid containing IFITM3 (I), but not in cells transfected with vector control (V). (**b**) Virus titers in the supernatants of cells transfected with vector control or IFITM3 containing plasmid at 24 h after virus infection. An average of a 5.4-fold decrease was observed in IFITM3-transfected cells compared to vector controls. (**c**) CCK-8 cytotoxicity assay was used to compare the difference in cell viability between QCXIP vector control and IFITM3-HA over-expressing MARC-145 cells. A *p*-value < 0.05 was considered statistically significant.

Western blotting analysis confirmed the expression of exogenous IFITM3 in cells transfected with a plasmid containing IFITM3-HA (Figure 1a), but not in cells transfected with the pQCXIP vector control (Figure 1a). Virus titers in the supernatants of cells harvested at 24 hpi showed an average of 5.4-fold decrease in IFITM3-HA-transfected cells compared to vector controls (*p* > 0.05) (Figure 1b). Cytotoxicity assay showed that there were no significant differences in cell viability between the vector control group and the IFITM3 over-expressing group (*p* > 0.05) (Figure 1c). The mean absorbance values of the vector control group and the IFITM3-over-expressing group were 1.48 and 1.32, respectively (Figure 1c). These results suggest that the over-expression of the IFITM3 protein reduces PRRSV replication.

### 3.2. Knockdown of IFITM3 by siRNA Enhances PRRSV Replication

**Figure 2:**
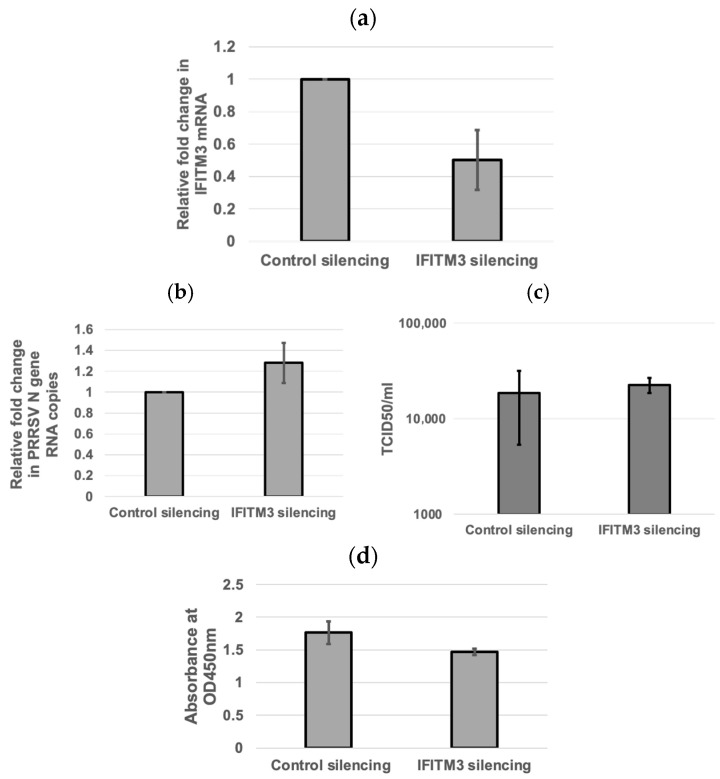
The silencing of IFITM3 slightly enhances PRRSV replication. (**a**) qRT-PCR showing the silencing of IFITM3. The averages and standard deviations of three replicates are shown. (**b**) qRT-PCR showing the viral RNA copies in control silencing and IFITM3 silencing RNA-transfected cells. The averages and standard deviations of three replicates are shown. (**c**) A slightly enhanced TCID_50_ virus titer (1.22-fold increase) in IFITM3 silencing RNA-transfected cells compared to control silencing RNA-transfected cells was observed. The averages and standard deviations of three replicates are shown. (**d**) The difference in cell viability was compared between the negative control siRNA and IFITM3 siRNA-transfected MARC-145 cells. The graph shows the mean absorbance values read at 450 nm between the two groups. A *p*-value < 0.05 was considered significant.

To validate the knockdown of endogenous IFITM3, qRT-PCR was performed to analyze the fold change in IFITM3 mRNA expression in IFITM3-silenced MARC-145 cells as compared to control silencing RNA. qRT-PCR confirmed the silencing of IFITM3, with an average of 50% knockdown efficiency (Figure 2a). The qRT-PCR and TCID_50_ assays were performed to quantify the viral RNA copies and supernatant virus titers in control silencing and IFITM3 silencing RNA-transfected cells. An average of 1.28-fold increase in viral RNA copies (*p* > 0.05) and a 1.22-fold increase in virus titers (*p* > 0.05) were observed in IFITM3 silencing RNA-transfected cells compared to control silencing RNA-transfected cells (Figure 2b,c). There were no significant differences (*p* > 0.05) in cell viability between control silencing RNA and IFITM3 silencing RNA (Figure 2d). The mean absorbance values of the negative control silencing group and IFITM3 silencing group were 1.76 and 1.47, respectively (Figure 2d). This shows that the knockdown of IFITM3 slightly enhanced virus replication.

### 3.3. Inverse Correlation Between IFITM3 Expression Level and Virus Replication Efficiency

**Figure 3:**
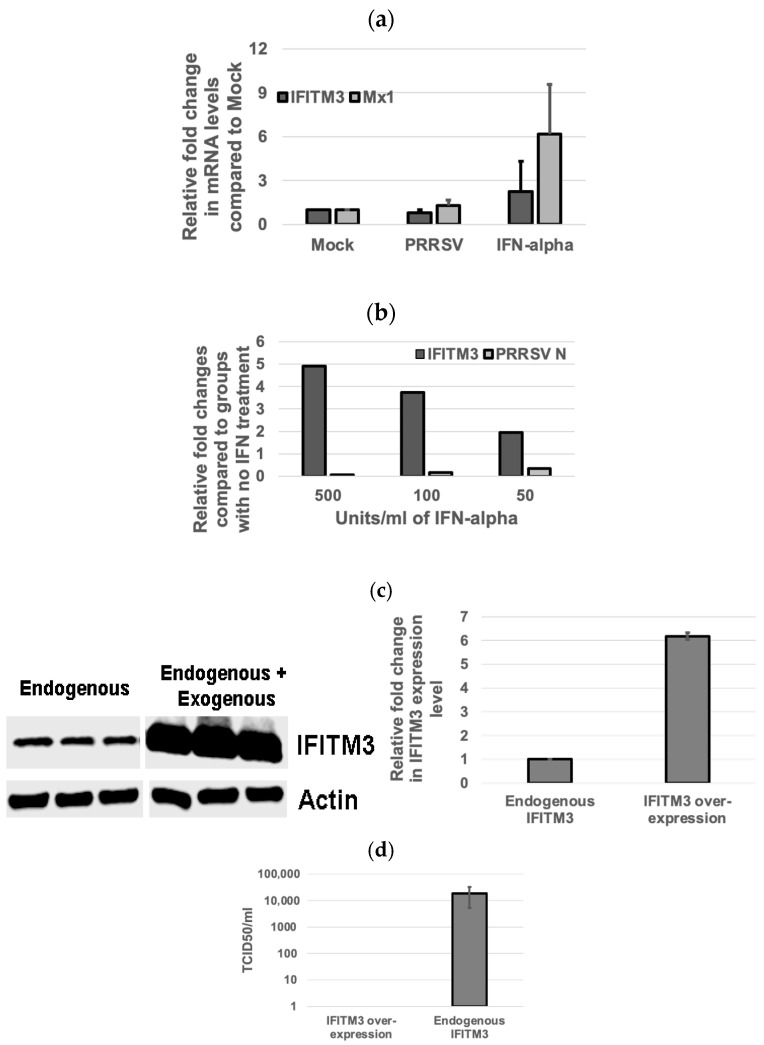
The IFITM3 expression level is inversely correlated to PRRSV replication efficiency. (**a**) IFN-alpha induces IFITM3 expression. Relative fold changes in IFITM3 and Mx1 gene expression in PRRSV 23,983-infected groups when compared to mock-treated groups are shown. More abundant Mx1 as compared to IFITM3 mRNA is induced by IFN-alpha. Representative data show the average and standard deviation of three replicates. (**b**) IFN-alpha inhibits PRRSV infection in a dose-dependent manner. Real-time RT-PCR shows the relative fold changes in IFITM3 and PRRSV N mRNA gene expression in IFN-alpha-treated groups at different concentrations when compared to non-treated control groups. Representative data show the average of two replicates. (**c**) The over-expression of IFITM3 results in 6.2-fold increase in the total IFITM3 protein level compared to the endogenous IFITM3 expression level. (**d**) No virus titer was detected in cells over-expressing IFITM3 while 1.9 × 10^4^ TCID_50_ titer was detected in cells only expressing the endogenous level of IFITM3.

When compared to the non-treated control group, PRRSV infection in MARC-145 cells upregulated Mx1 gene expression but not that of IFITM3 (Figure 3a). IFN-alpha treatment induced the upregulation of mRNA gene expression of both IFITM3 and Mx1 as compared to the control group (Figure 3a). At a 50 U/mL IFN-alpha concentration, the mean fold change in IFITM3 gene expression as compared to IFN-alpha-untreated cells was 1.94 (Figure 3b). At a 100 U/mL concentration of IFN-alpha, the mean fold change in IFITM3 gene expression was 3.73 (Figure 3b). At 500 U/mL concentration of IFN-alpha, the mean fold change in IFITM3 expression was 4.91 (Figure 3b). A dose-dependent increase in IFITM3 mRNA level in IFN-alpha-treated MARC-145 cells was detected. As compared to the IFN-alpha-untreated group infected with PRRSV, the mean fold changes in the PRRSV N gene transcripts upon treatment with IFN alpha at 50 U/mL, 100 U/mL, and 500 U/mL were 0.34, 0.16, and 0.07, respectively (Figure 3b). A dose-dependent decrease in PRRSV gene transcript in IFN-alpha-treated MARC-145 cells was observed. These results showed an inverse correlation between IFITM3 expression and PRRSV replication in IFN-alpha-treated MARC-145 cells.

Western blotting analysis showed that IFITM3 over-expression increased the total IFITM3 protein by 6.2-fold when compared to the endogenous IFITM3 level (Figure 3c). Accordingly, no virus was detected in cells over-expressing IFITM3 by TCID_50_ assay, while cells only expressing an endogenous level of IFITM3 had a virus titer of 1.9 × 10^4^/mL (Figure 3d). These data further confirm the inverse correlation between IFITM3 expression level and virus replication efficiency.

### 3.4. Amphotericin B Partially Restores PRRSV Replication in IFITM3-over-Expressing Cells

**Figure 4:**
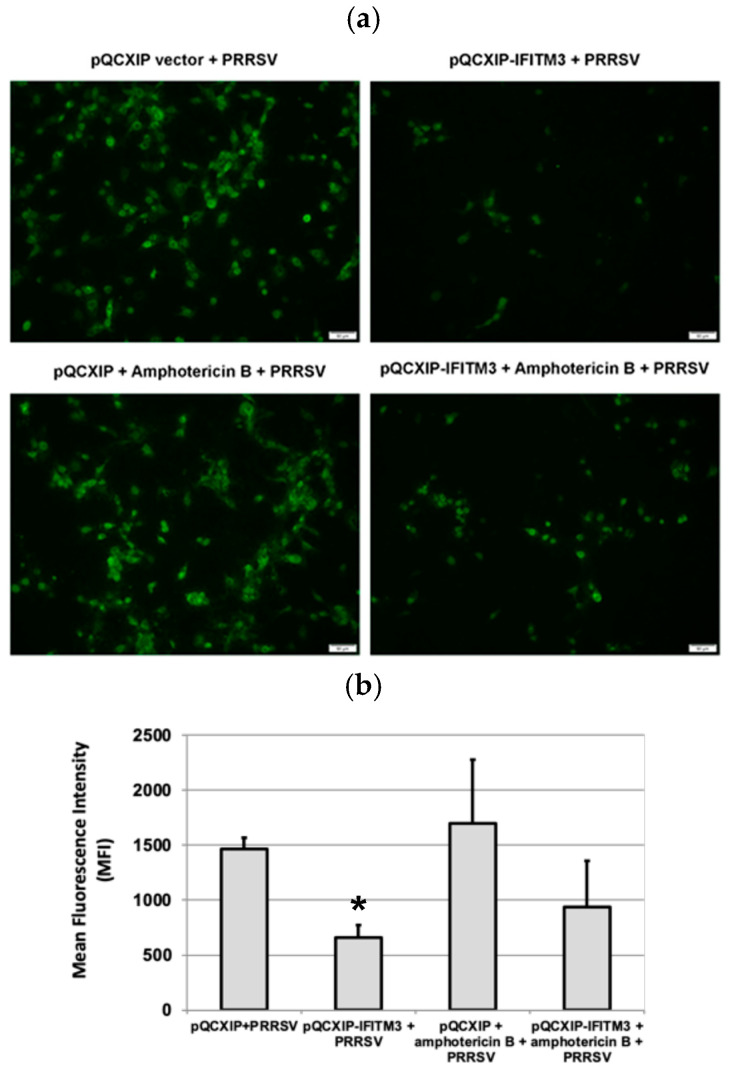
Amphotericin B treatment only partially restores PRRSV replication in cells over-expressing IFITM3. (**a**) Immunofluorescence staining of virus-infected cells in the presence or absence of amphotericin B treatment. Images were captured at 10× magnification. (**b**) Flow cytometry analysis of virus-infected cells in the presence or absence of Amphotericin B. * indicates *p* < 0.05 when compared to the pQCXIP + PRRSV group.

IFITM3 over-expression significantly reduced PRRSV infection in MARC-145 cells (Figure 4a). To quantitatively estimate the PRRSV infection in the Amphotericin B-treated and -untreated groups, flow cytometry was performed. Analysis of virus-infected cells in the presence or absence of Amphotericin B treatment confirmed that Amphotericin B partially restored the replication of PRRSV in cells over-expressing IFITM3 (Figure 4b). In the Amphotericin B-untreated group, a significant reduction in mean fluorescence intensity (*p* < 0.05) in the IFITM3-over-expressing cells as compared to vector control was observed (Figure 4b). This data further validates our observation shown in Figure 1.

### 3.5. Colocalization of PRRSV with Early and Late Endosome/Lysosome Markers at 3 Hpi

**Figure 5:**
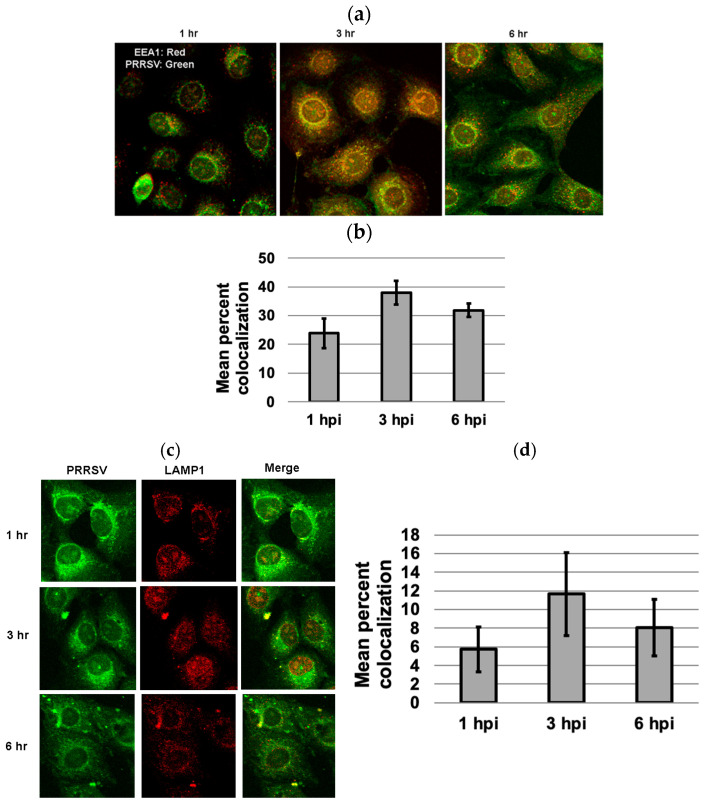
Colocalization of PRRSV with early endosome marker EEA1 and late endosome marker LAMP1 at 1, 3, and 6 h after virus infection. (**a**) Colocalization of PRRSV with EEA1 at different time points. (**b**) Mean percent colocalization was estimated by manual thresholding using Image J version 1.53. For each time point, mean percent colocalization was calculated by randomly selecting five cells for each microscopic field. A greater percentage of colocalization occurred with EEA1 at 3 hpi as compared to 1 or 6 hpi. (**c**) The colocalization of PRRSV with LAMP-1 at different time points. (**d**) Only limited colocalization between PRRSV and late endosome/lysosome marker LAMP1 was observed at 3 h after virus infection. The images were captured using a confocal microscope at 40× magnification.

To examine whether PPRSV enters the late endosomes, MARC-145 cells infected with PRRSV 23,983 for 1 h, 3 h, or 6 h were stained for either the early endosomal marker, EEA1, or the late endosome/lysosome marker, LAMP-1. While approximately 38% colocalization between PRRSV and EEA1 was observed at 3 hpi (Figure 5a,b), only 12% colocalization between PRRSV and LAMP-1 was observed at 3 h after virus infection (Figure 5c,d). Few colocalizations between PRRSV and EEA1 or LAMP1 were observed at 1 and 6 hpi (Figure 5).

### 3.6. Over-Expression of IFITM3 Does Not Significantly Impact Virus Entry

**Figure 6:**
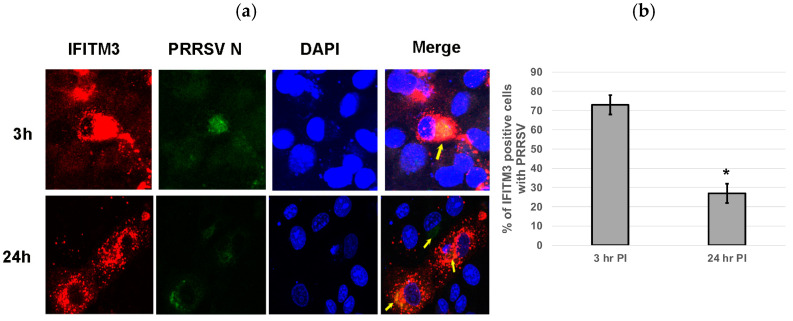
Over-expression of IFITM3 does not significantly impact virus entry. (**a**) Cells over-expressing exogenous IFITM3 contain PRRSV at 3 and 24 hpi. The colocalization of PRRSV with over-expressed IFITM3 was observed (yellow arrows). Red: IFITM3; green: PRRSV N; blue: DAPI. The images were taken at 40× magnification. (**b**) The percentage of PRRSV-positive cells was significantly lower (*p* < 0.0001) in IFITM3-transfected cells at 24 hpi than at 3 hpi. Quantification was performed by counting PRRSV-positive cells and IFITM3-HA-expressing cells obtained from five different microscopic fields. * indicates *p* < 0.0001.

Cells over-expressing exogenous IFITM3 were stained for PRRSV at 3 and 24 hpi. Colocalization of IFITM3 with PRRSV was observed at both time points (Figure 6a). An average 73% of IFITM3-expressing cells contained virus at 3 hpi, while only approximately 27% of IFITM3-expressing cells contained virus at 24 hpi (Figure 6b). Confocal images of IFITM3 over-expressing cells typically showed a few punctuate positive virus staining within isolated individual cells at 24 hpi (Figure 6a). indicative of inefficient cell–cell spread of virus.

### 3.7. Over-Expression of IFITM3 Reduces the Level of P-Akt and Increases the Ratios of LC3-II/LC3-I

**Figure 7:**
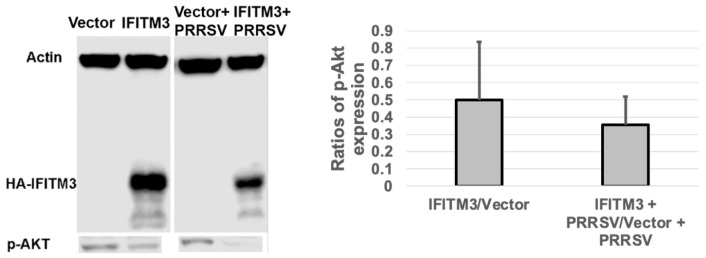
The over-expression of IFITM3 reduces the level of phosphorylated Akt by 50% compared to the vector control. A representative Western blot image shows the expression level of p-Akt. The averages and standard deviations of three replicates are shown in the graph. A significant decrease in the level of p-Akt was observed in IFITM3-over-expressing and PRRSV-infected cells compared to vector-transfected and PRRSV-infected cells (*p* < 0.05).

To examine whether IFITM3 over-expression affects the level of p-Akt and the ratios of LC3-II/LC3-I, we performed Western blot analysis. As shown in Figure 7, IFITM3 over-expression reduced the level of p-Akt by approximately 50% compared to vector control-transfected cells. Furthermore, a significant decrease (*p* < 0.05) in p-Akt level was observed in PRRSV-infected cells over-expressing IFITM3 compared to PRRSV-infected cells transfected with vector control. An approximately 65% reduction in p-Akt was observed in PRRSV-infected cells over-expressing IFITM3 compared to PRRSV-infected cells transfected with vector control (Figure 7).

**Figure 8:**
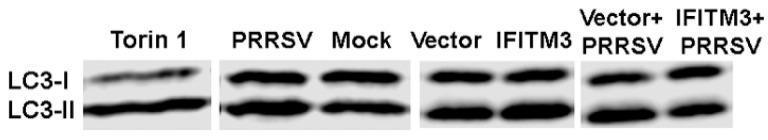

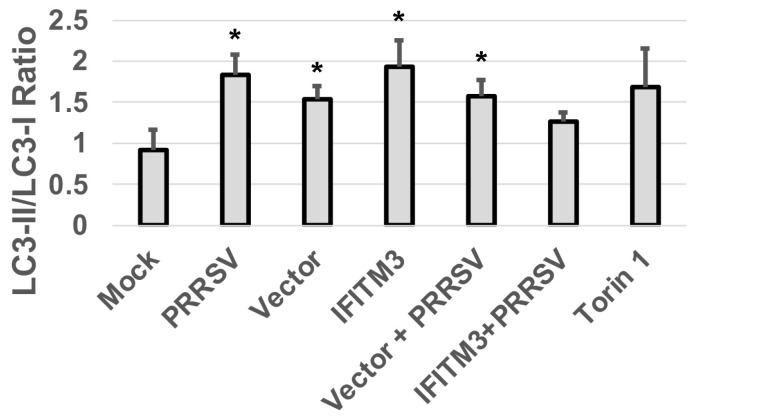
The over-expression of IFITM3 increases the ratio of LC3-II/LC3-I by 128% compared to vector control. A representative Western blot image shows the expression level of LC3-I and LC3-II. The averages and standard deviations of three replicates are shown in the graph. * Indicates significant differences compared to mock (*p* < 0.05).

Similarly, a slightly increased LC3II/LC3-I ratio was observed in IFITM3 over-expressing cells compared to vector control cells (Figure 8). A slight decrease in LC3II/LC3-I ratio was observed in PRRSV-infected cells over-expressing IFITM3 than PRRSV-infected cells transfected with vector control (Figure 8). PRRSV infection, vector control transfection, and IFITM3 over-expression all induced a significantly higher ratio of LC3-II/LC3-I than mock cells (*p* < 0.05).

## 4. Discussion

IFITM3 is an interferon-induced antiviral gene and partly contributes to the antiviral effect of interferon. Indeed, IFITM3 has been shown to restrict the replication of a diverse array of enveloped RNA viruses including PRRSV [2,14,29]. We have shown here that the over-expression of IFITM3 reduced PRRSV replication in MARC-145 cells, which is consistent with the previous observation made by others [22]. Similarly, the silencing of IFITM3 enhanced PRRSV replication as shown by both our study and Zhang et al. [22]. The observed virus replication enhancement effect in our study is lower than what has been described previously [22], which could be due to the relatively low knockdown efficiency (an average of 50%) and the high MOI of the virus used in our study. This is supported by the recent report showing that IFITM3 is effective in restricting the lower threshold of virus infection [30]. Collectively, both the over-expression and RNA silencing of IFITM3 data suggest its antiviral effect against PRRSV.

IFITM3 is constitutively expressed in both MARC-145 cells and porcine alveolar macrophages (PAMs). Our study and others [22] have shown upregulation of the IFITM3 protein upon IFN-α treatment, which is positively correlated with reduced PRRSV replication. PRRSV upregulated IFITM3 expression in PAMs at 24 hpi [22]. Interestingly, we observed that PRRSV infection reduced IFITM3 gene expression in MARC-145 cells at 24 hpi. This discrepancy could be due to different cell types used in each study.

It is generally believed that IFITM3 decreases membrane fluidity thereby inhibiting virus-cell membrane fusion and cytosolic release [6]. Studies using single-virus fusion assays have shown that IFITM3 restricts virus membrane fusion [31,32]. One earlier study reported that PRRSV is associated with early endosomes only, and not late endosomes [21]. Similarly, we found that very few PRRSV colocalized with LAMP-1, a marker of late endosome and lysosome, during the first 6 hpi. PRRSV colocalized with the early endosome marker EEA1 at 3 hpi, but most disappeared from the early endosome at 6 hpi. IFITM3 is known to restrict virus membrane fusion and trap the virus in the late endosome for degradation [23,33]. Amphotericin B treatment has been shown to restore infection of many viruses that are restricted by IFITM3 [32,34]. In a recent study, Amphotericin B reversed the IFITM3-mediated entry-restriction of several pseudovirus particles tested, including SARS-CoVpp and IAVpp, but not LASVpp [35]. Amphotericin B is an antifungal drug known to restore membrane fluidity, thereby reversing the effect of IFITM3-mediated virus restriction [34]. It forms leak ion channels in the cell membrane and has also been shown to associate with cholesterol in lipid rafts [23]. Our observation suggests that IFITM3 may restrict PRRSV in a mechanism different from SARS-CoV and IAV [35] since only 12% of virus was detected in the late endosome. This observation is also consistent with our data showing that Amphotericin B treatment only partially restores PRRSV replication. Interestingly, Zhang et al. have shown that PRRSV colocalizes not only with early endosomes but also late endosomes/lysosomes [22]. The discrepant results could be due to different experimental conditions used in each study. Zhang et al. have also shown that IFITM3 does not restrict PRRSV attachment, entry, and internalization into endosomes, but inhibits virus membrane fusion via cholesterol induction in cellular vesicles [22].

Suddala et al. showed that IFITM3-expressing cells are not infected by IAV at 12 hpi, but adjacent cells not expressing IFITM3 are infected, suggesting that this antiviral activity of IFITM3 is not due to conditioned medium [36]. Our results showed that IFITM3-expressing cells do contain PRRSV at both 3 h and 24 h pi, suggesting that a potentially different mechanism mediates the antiviral activity against PRRSV rather than IAV. Suddala et al. suggest that IFITM3 accumulation at the sites of virus fusion prevents virus release into the cytoplasm and subsequent virus replication [36]. We observed an average of 73% of IFITM3-expressing cells contain PRRSV at 3 hpi, indicating that the over-expression of IFITM3 has little effect on virus entry. However, at 24 hpi, only 27% of IFITM3-expressing cells contained PRRSV. Our study also showed that PRRSV in IFITM3-over-expressing MARC-145 cells exhibited a punctuated staining pattern in isolated individual cells at 24 hpi (Figure 6a). These observations suggest that efficient cell–cell spread of the virus may be affected by the over-expression of IFITM3, which has also been shown by Zhang et al. [22]. Earlier studies have shown that PRRSV upregulates the formation of tunneling nanotubes (TNTs) to facilitate the intercellular spread of PRRSV [37]. Hence it is tempting to speculate that IFITM3 over-expression may affect the cellular spread of the virus by limiting the formation of TNTs. Further studies are needed to uncover the impact of IFITM3 over-expression on the formation of TNT upon PRRSV infection. Overall, our data support a role for IFITM3 in restricting PRRSV post its entry into cells.

The Phosphatidylinositol 3-kinase (PI3K)-dependent Akt signaling pathway is involved in cell survival, cell proliferation, and differentiation. The pro-viral effect of the PI3K/Akt pathway on the replication of many viruses including PRRSV has been reported previously [38]. It is reasonable to speculate that the reduced p-Akt level in IFITM3-over-expressing cells may also partially contribute to the reduced virus replication observed here. It has been reported that the over-expression of IFITM3 or other ISGs such as GBP1 can induce autophagy [39,40], which may restrict virus replication [39]. An increased LC3-II/LC3-I ratio in IFITM3-over-expressing MARC-145 cells rather than vector control cells may also partially contribute to the reduced PRRSV replication. In a separate study, we have observed that the over-expression of IFITM3 in H1299 cells induced autophagy and enhanced Seneca virus A replication [41]. Akt is known to regulate autophagy [42], which is supported by our observation showing the correlation between p-Akt and autophagy marker (LC3-II/LC3-I ratio). Interestingly, several previous studies have reported that autophagy sustained or enhanced PRRSV replication [42,43,44,45]. Autophagy can serve as a double-edged sword during virus infections [46]. Future studies are needed to examine the role of cellular changes due to the non-physiological level of IFITM3 over-expression and their contribution to the replication efficiency of other viruses in different cell types.

## 5. Conclusions

In conclusion, over-expression of IFITM3 reduced PRRSV infection and may restrict PRRSV at the post-entry steps since amphotericin B treatment did not completely rescue PRRSV infection. The silencing of endogenous IFITM3 increased PRRSV RNA copies and virus titers. Furthermore, an inverse correlation between the IFITM3 level and PRRSV gene copies was observed. Further study is needed to better understand the mechanisms by which IFITM3 restricts PRRSV replication in MARC-145 cells and porcine alveolar macrophages (PAMs).

## Figures and Tables

**Table 1:** Primer sequences of IFITM3, PRRSV N, and the house-keeping gene (beta-actin).

Gene	Forward Primer (5′-3′)	(Reverse Primer (5′-3′)
beta-actin	TTGCTGACAGGATGCAGAAGGAGA	ACTCCTGCTTGCTGATCCACATCT
IFITM3	GGTCTTCGCTGGACACCAT	TGTCCCTAGACTTCACGGAGTA
PRRSV N	GTCAATCCAGACCGCCTTTA	GATCAGGCGCACAGTATGAT
Mx1	TTTTCAAGAAGGAGGCCAGCAA	TCAGGAACTTCCGCTTGTCG

## Data Availability

The original contributions presented in this study are included in the article. Further inquiries can be directed to the corresponding author.

## Abbreviations

The following abbreviations are used in this manuscript:

IFITM3	Interferon-induced transmembrane protein 3
PRRSV	Porcine reproductive and respiratory syndrome virus
ISGs	Interferon-stimulated genes
ZMPSTE24	Zinc metallopeptidase STE24
IAV	Influenza A virus
MEFs	Mouse embryonic fibroblasts
vRNPs	Viral ribonucleoproteins
SARS-CoV	Severe acute respiratory syndrome coronavirus
LASVpp	Lassa virus pseudovirus
PI3K	Phosphatidylinositol 3-kinase
GBP1	Guanylate binding protein 1
TNTs	Tunneling nanotubes
